# Shift work-like patterns effect on female and male mouse behavior

**DOI:** 10.1016/j.nbscr.2022.100082

**Published:** 2022-10-08

**Authors:** Gareth Banks, Patrick M. Nolan, Nora Bourbia

**Affiliations:** a MRC Harwell Institute, Harwell Science and Innovation Campus, Harwell, Oxfordshire, OX11 0RD, UK; b UK Health Security Agency, Harwell Campus, Chilton, Didcot, OX11 0RQ, UK

**Keywords:** Circadian rhythm, Mouse behavior, Shift-work

## Abstract

Shift work (work outside of standard daylight hours) is common throughout the Western world. However, there are notable health consequences to shift work, including increased prevalence of mental health and sleep disorders in shift worker populations. Therefore, the health and wellbeing of shift workers is a public health concern that needs to be addressed. Here we investigate the effects of two separate light induced shift work-like patterns on male and female mouse behaviour (anxiety-like, exploration, marble burying, startle reflex and circadian rhythms). After 6 weeks of shift-like disruptions patterns, animals displayed no behavioral differences in exploration, marble burying and startle reflex. Interestingly however, we identified sex specific and disruption specific effects in light aversion and wheel running activities. Notably, analysis of the activity patterns of animals in disruptive conditions demonstrated that they maintained a degree of rhythmicity through the disruption period, which may explain the lack of behavioral differences in most behavioral tests.

## Highlights

• Photoperiod shifts disturb the weekly rhythmicity in both female and male mouse.
• Alternated photoperiod shift decreases the voluntary movement activity in mouse.
• Forwarded photoperiod shift reduces the natural aversion of male mice to light.
• Female mice have a stronger daily rhythm.
• Female mice have a higher voluntary wheel running activity.

## 1 Introduction

To adapt to the nychthemeron in Earth, virtually all organisms have developed a 24 h-period circadian rhythm synchronised by the day to night alternation of light and dark. The suprachiasmatic nucleus (SCN) within the hypothalamus of the brain is the master pacemaker of this rhythmicity and receives direct input from the retina of the eye. Because the light is the main cue (known as a zeitgeber) synchronising the circadian rhythms, ill-timed light exposure can induce circadian misalignment. For instance, light pollution and shift-working disrupt circadian rhythms(Navara and Nelson, 2007) and this disruption has a demonstrably negative impact on human health(Rajaratnam and Arendt, 2001). Shift work is defined as work performed outside standard daylight hours (7/8 a.m.–5/6 p.m.). This includes night and rotation shifts. With 21% of the European working population being shift-workers in 2015(European Foundation for the Improvement of Living and Working Conditions and Eurofound, 2017), the well-being of shift-workers is an important public health topic as shift-workers are widely reported to have a poorer health(Bara and Arber, 2009). At the neurologic and psychiatric level, shift-workers report sleep disturbances(Fekedulegn et al., 2016; Lajoie et al., 2015) which are themselves associated with impaired vigilance and increase of risk of accidents(Boivin and Boudreau, 2014). These symptoms are the manifestation of shift work sleep disorder(Di Milia et al., 2013) recognised by the international classification of sleep disorders(Sateia, 2014). Up to 32.1% of shift workers develop shift work sleep disorder making them more susceptible to sleepiness with an increased risk of accidents which is a public safety concern. Furthermore, the association between shift-working and presence of anxiety, depression and the increase of risk of dementia is documented in the literature(Bokenberger et al., 2018; Kalmbach et al., 2015; Lee et al., 2016; Togo et al., 2017) Notably the type of shift-work affects the sexes differentially: men report poorer health and anxiety/depression after more than 4 years of night shift work while women report the same mental health issues following more than 2 years of varied shift pattern but not in night-shift(Bara and Arber, 2009).

While the link between circadian rhythms and health is established, the mechanism of how shift-work might affect health is poorly understood. The use of mice to study shift-work allows a control of the environmental factors including the genetic background, and also focuses on the direct effect of the shift-work pattern. Studying shift-work in rodents has been achieved by placing the rodent in a slow rotating wheel during the light time(Salgado-Delgado et al., 2010) or shifting the photoperiod(Deibel et al., 2014; McGowan and Coogan, 2013; Okuliarova et al., 2016). McGowan and Coogan (2013) have shown that rapidly rotating shift work patterns (by either alternating the 12 h:12 h cycle or shifting 8-h forward or backward the cycle every two days for six days followed by 2 days of constant darkness for 6 weeks) increases the anxiety-like behaviour assessed by the open field test. Other studies have found similar results, demonstrating shift-work disruptive effects on memory and on mood(Loh et al., 2010; McDonald et al., 2013; Okuliarova et al., 2016). However, such studies often use outbred or mixed strain animals, which are not comparable to the inbred lines often used in behavioural studies. Furthermore, these studies often use a limited range of behavioural tests, meaning that the range of effect of shift-work is not fully characterised. Finally, previous studies have also often only studied the effect of shift-working on a single sex, leaving any potential sex specific effects uncharacterised. Here we use the light induced shift-work paradigm outlined in McGowan and Coogan (2013) to study the effect of long-lasting changes (2 weeks after re-entrainment to the initial light-dark cycle) from long-term shifting on a range of downstream behaviours. Notably while the negative impact of circadian disruption is often reported as transient(Bedrosian et al., 2013)McGowan and Coogan report behavioural changes which persist following reentrainment following their disruption protocols. Here we characterise the effect of the disruptive light cycles to animal behaviour and highlight sex specific effects in downstream behaviours.

## 2 Materiel and methods

### 2.1 Animals

The experiments were performed with adult female and male C57BL/6 J (B6J) mice at the age of 3 months and bred at the Mary Lyon Centre in Harwell (UK) and under the guidance issued by the Medical Research Council and Home Office Project License 30/3206, with local ethical approval. Animals were housed in Blue Line IVC cages (Techniplast) (base size: 35 × 14 × 19cm LxHxW), containing Eco-Pure Lab Animal Bedding (Datesand) and FDA Paper Shavings (Datesand). RM3 food (Dietex) and water was available ad libitum. All efforts were made to limit distress and to use only the number of animals necessary to produce reliable scientific data. Mice were initially grouped housed in a 12-h light/dark cycle with food and water access ad libitum until the beginning of the photoperiod shift. Tunnel or cupping methods of handling were used for routine animal checks and cage changes, while the tail handling method were used to transfer animals to the experiment tasks.

### 2.2 Photoperiod shift

A total of 60 mice has been used. 30 mice per sex randomly assigned in 3 groups of 10 animal each: control, alternate and forward according to the Table 1. They were singly housed in cages with running wheels to record their voluntary wheel running activity as described in(Banks and Nolan, 2011). The rhythm amplitude, interdaily stability (IS), intradaily variability (IV) and wheel running activity were recorded for the complete duration of the protocol. IS measures the day-to-day reproducibility of rest/activity cycles whereas IV is a measure of the fragmentation of activity rhythms(Brown et al., 2019).Circadian analysis during the disruption period was performed using Clocklab (Actimetrics). At the end of the photoperiod shift, the female mice were grouped housed while males were kept singly housed, both in individually ventilated cages for at least 14 days to re-entrain to a standard 12 h light cycle before starting behavioural tests. The aim of the period of 14 days re-entrainment is to exclude potential artefact effect of the animals being in different circadian phases at the end of the shift-work paradigms, and to assess whether shift-work paradigms induce behavioural differences lasting beyond re-entrainment to normal cycles.

**Table 1:** Shift work-like photoperiod patterns.

	+ Lights on; – Lights off		
	Control	Forward	Alternating
Day 1	+07:00–19:00	+17:00–05:00	+07:00–19:00
Day 2	+07:00–19:00	+17:00–05:00	+07:00–19:00
Day 3	+07:00–19:00	+23:00–11:00	+07:00 (Constant light for 24 h)
Day 4	+07:00–19:00	+23:00–11:00	−07:00 + 19:00
Day 5	+07:00–19:00	+7:00–19:00	−07:00 + 19:00
Day 6	+07:00–19:00	+7:00–19:00	−07:00 (Constant darkness for 24 h)
Day 7	Constant darkness	Constant darkness	Constant darkness
Cycle repeated 5 times			

### 2.3 Behavioral tests

Animal were assigned behavioural tests in a randomised fashion and all tests were performed blind to the shift-work paradigms. The light dark box and open field tests were automated using Ethovision software. The prepulse inhibition test was automated using Med Associates PPI chambers. All tests were performed during the afternoon and corresponding to the light phase for the mice. The order of the behavioral test was: Light Dark box, Open Field, Acoustic startle and Pre-pulse inhibition (PPI), and Marble Burying. For technical and logistical reasons, we were unable to run the complete cohort of animals through all phenotyping tests. However, if an animal was not able to be run through a specific test, it was removed from all subsequent tests, meaning that all animals within a test had the same test history.

#### 2.3.1 Light dark box

To assess anxiety-like behaviour the mouse is placed inside a 40 cm × 40 cm x 40 cm plastic box, equally divided in two compartments, for 5 min. One compartment is exposed to 200 lux and the second compartment is dark without direct light exposure, with light exposure present only through the entrance of the dark compartment from the light compartment (a 3 cm × 3 cm open door). The mouse is initially placed in the dark compartment and can freely access to both compartments using a 3 cm × 3 cm door in the middle of the box. The time spent in each compartment, the number of entries and the latency to the first entry to the light are measured.

#### 2.3.2 Open field

To assess exploratory and anxiety-like aspects of behavior, the mouse is placed in a 45 cm × 45 cm arena exposed to 200 lux for 10 min. The arena is divided in two zones of exploration: the periphery and the centre of the arena (20 cm × 20 cm). The mouse is initially placed at the periphery of the arena. The time spent on both zones, the number of entries and the latency to the first entry in the centre area and the velocity of the mice moving in both zones are measured.

#### 2.3.3 Acoustic startle and pre-pulse inhibition (PPI)

Acoustic startle and pre-pulse inhibition were assessed using acoustic startle chambers (Med Associates). To assess the startle response, a 5-min acclimation period was followed by five 10 ms 120 db white noise pulses (with an inter-trial interval of 20–30 s). Reponses were expressed in arbitrary units and averaged. To assess PPI, a 120 db white noise pulse was preceded by either a 70 or a 75 db prepulse, with a 50 ms interval between the pulse and the prepulse. The amount of PPI is expressed as a percentage of baseline startle.

#### 2.3.4 Marble burying

To assess species-specific digging behavior that could reveal some compulsive-like behaviour(Angoa-Pérez et al., 2013), a regular IVC cage (base size: 35 × 14 × 19cm LxHxW) is filled with the equivalent of three cages of sawdust bedding and nine marbles are place on the top of the bedding. The mouse is initially placed at the corner of the cage. After 15 min, the mouse is taken back to its home cage and the numbers of buried marbles are counted.

### 2.4 Statistical test

2-way ANOVA followed by Tukey’s multiple comparisons test if 2-way ANOVA would show either a sex difference and/or a shift-work difference was used to analyse photoperiod shift data, Light-Dark box, Marble burying, Open Field and Startle reflex and Prepulse Inhibition tests to compare shift-work paradigms and sex difference. Correlation analyses were performed using Pearson Correlations, using the Benjamini-Hochberg procedure for multiple test corrections with sex used as a categorisation factor.

## 3 Results

### 3.1 Photoperiod shift

**Fig. 1:**
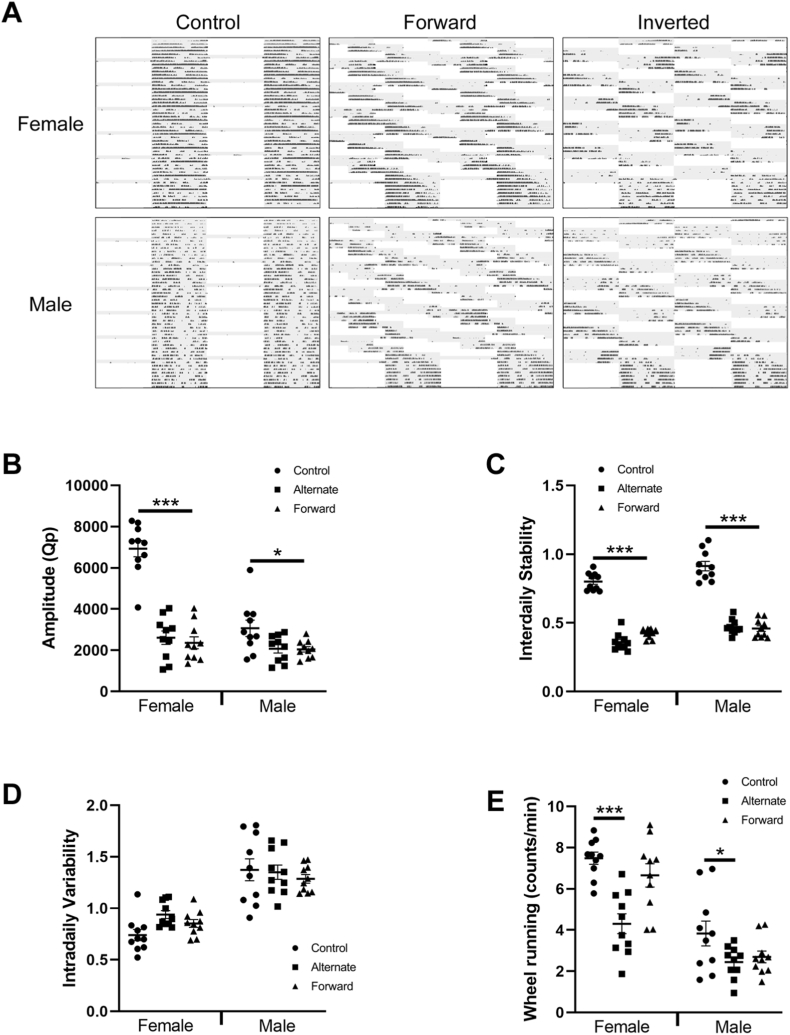
Circadian analysis of animals during the disruption period. A. Example of an actogram of the control and both forward and inverted (shift work-like) photoperiod shift in male and female mouse. B. The circadian amplitude of animals during the disruption period. C. The interdaily stability (IS) of animals during the disruption period. D. The intradaily stability (IV) of animals during the disruption period. E. The wheel running activity of animals during the disruption period. *p < 0.05, ***P < 0.001.

Periodogram analysis of wheel running activity during photoperiod shifts suggested that animals in both the alternate and forward lighting conditions shifted their activity rhythms to match the pattern of activity of the disruptive light cycle. Visual inspection of actograms revealed that in the alternate and forward conditions, animals alternated between periods of high activity (when they were in phase with the light cycle) and low activity (when they were out of phase) (Fig. 1A).

In order to characterise the activity rhythms of the animals, the rhythm amplitude, interdaily stability (IS), intradaily variability (IV) and wheel running activity were recorded for the complete duration of the protocol. Prior to analysing the effect of altered photoperiod, we compared male and female control animals to identify sex differences in activity rhythms under non-disruptive conditions. Here we found that compared to males, female animals show a significantly higher amplitude (male = 3070 ± 396, female = 6930 ± 393; F[1,18] = 47.03 p = 0.0001) (Fig. 1B), a significantly lower IV (male = 1.37 ± 0.05 counts/min, female = 0.75 ± 0.02 counts/min; F[1,18] = 23.48 p = 0.0001) (Fig. 1D) and significantly higher wheel running activity (male = 3.83 ± 0.6, female = 7.48 ± 0.29; F[1,18] = 28.64 p = 0.0001) (Fig. 1E). IS was lower in female animals compared to males, but this did not reach significance (male = 0.879 ± 0.02, female = 0.798 ± 0.01; F[1,18] = 4.28 p = 0.0532) (Fig. 1C).

Analysis of activity rhythms of disrupted animals over the complete duration of the protocol, demonstrated that male animals under forward or alternate photoperiod shifts showed a significant reduction in amplitude (control = 3070 ± 396, forward = 2048 ± 131, alternate = 2075 ± 161; F[2,27] = 4.72 p = 0.017) (Fig. 1B) and IS (control = 0.879 ± 0.02, forward = 0.362 ± 0.01, alternate = 0.439 ± 0.02; F[2,27] = 128.69 p = 0.0001) (Fig. 1C) but no significant difference in IV (control = 1.38 ± 0.06, forward = 1.27 ± 0.03, alternate = 1.31 ± 0.04; F[2,27] = 0.46 p = 0.639) (Fig. 1D). Wheel running activity was significantly lower for male animals under alternate lighting conditions (control = 3.83 ± 0.6 counts/min, alternate = 2.44 ± 0.25; F[2,27] = 3.21 p = 0.046), but was not significantly affected under forward lighting conditions (control = 3.83 ± 0.6 counts/min, forward = 2.69 ± 0.28; F[2,27] = 3.21 p = 0.11) (Fig. 1E). Similarly to males, female animals under forward or alternate photoperiod shifts showed a significant reduction in amplitude (control = 6930 ± 393, forward = 2366 ± 289, alternate = 2610 ± 325; F[2,27] = 57.3 p = 0.0001) (Fig. 1B), IS (control = 0.798 ± 0.01, forward = 0.364 ± 0.02, alternate = 0.323 ± 0.01; F[2,27] = 128.69 p = 0.0001) (Fig. 1C), no significant difference in IV (control = 0.752 ± 0.03, forward = 0.858 ± 0.03, alternate = 0.922 ± 0.03; F[2,27] = 3.22 p = 0.068) (Fig. 1D) and significantly lower wheel running activity in the alternate condition (control = 7.48 ± 0.29 counts/min, alternate = 4.31 ± 0.48; F[2,27] = 12.77 p = 0.0001) but not in the forward condition (control = 7.48 ± 0.29 counts/min, forward = 6.65 ± 0.56; F[2,27] = 3.21 p = 0.35) (Fig. 1E).

### 3.2 Anxiety-related phenotypes

#### 3.2.1 Light-dark box

**Fig. 2:**
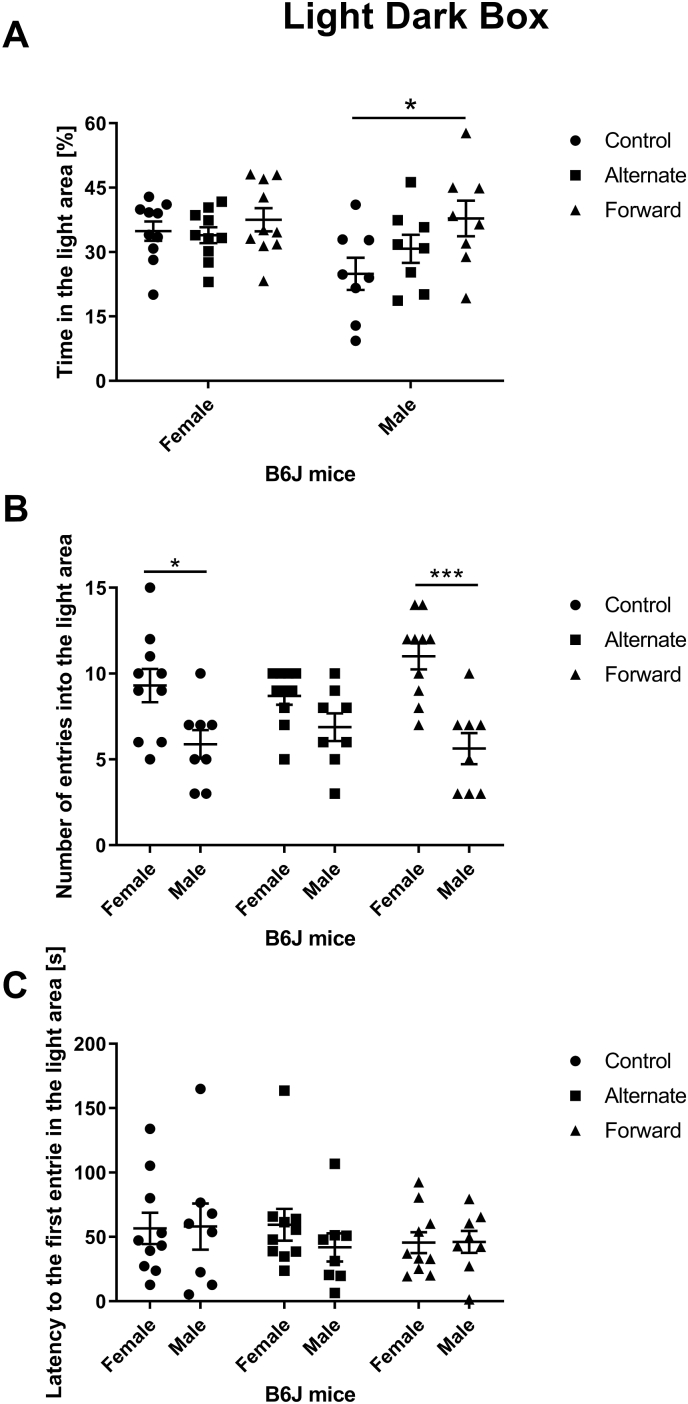
5 min in the Light dark box test with three shift-work paradigms: control (black circle), alternate (black square) and forward (black triangle) of B6J female and male adult mice. A. Percentage of time spent in the light area. B. Latency to the first entry in the light area. C. Number of entries in the light area. *p < 0.05, ***P < 0.001.

Only the forward shift-work paradigm induces an increase of the time spent in the light area in males compared to controls but no difference in females in either shift-work paradigm (2-way ANOVA; Shift-work paradigm effect: F[2,48] = 3.606, p = 0.0348, sex difference: F[1,48] = 3.120, p = 0.0837; interaction: F[2,48] = 1.552, p = 0.2224. Tukey’s multiple comparisons test: male control vs male forward: p = 0.0143, n = 8 to 10 per group) (Fig. 2A). The shift-work paradigms do not affect the number of entries in the light area (2-way ANOVA; Shift-work paradigm effect: F[2,48] = 0.4048, p = 0.0979, sex difference: F[1,48] = 30.04, p < 0.0001; interaction: F[2,48] = 2.627, p = 0.4636; n = 8 to 10 per group) (Fig. 2B) nor the latency to the first entries in the light area (2-way ANOVA; Shift-work paradigm effect: F[2,48] = 0.4607, p = 0.6336, sex difference: F[1,48] = 0.2785, p = 0.6001; interaction: F[2,48] = 0.3966, p = 0.6748; n = 8 to 10 per group) (Fig. 2C).

**Fig. 3:**
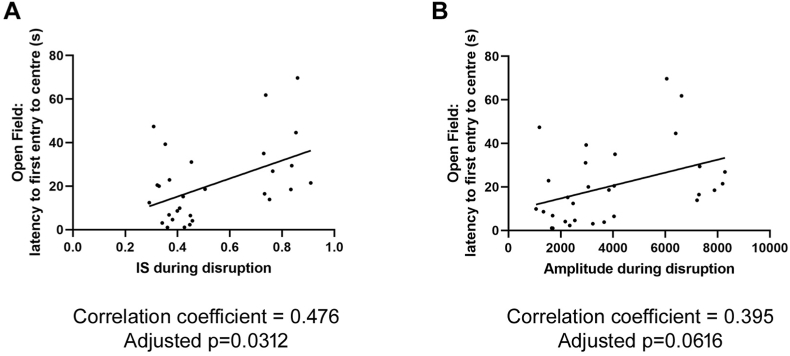
Correlations between circadian disruption parameters and downstream behaviors in female mice. A. A significant correlation between IS during disruption and the latency to enter the centre of the open field. B. A near significant correlation between circadian amplitude during disruption and the latency to enter the centre of the open field.

However, a sex difference is significantly present in the number of entries in the light area when comparing female and male WT controls (Tukey’s multiple comparisons test: p = 0.0485) and female and male forward shifted (p < 0.0001) mice (2-way ANOVA; sex difference: F = 30.04, p = 0.0003; Tukey’s multiple comparisons test, n = 8 to 10 per group) (Fig. 2B).

#### 3.2.2 Open field

There is no difference between the shift-work paradigms and controls of either sex for the time spent in the centre of the arena (2-way ANOVA; Shift-work paradigm effect: F[2,54] = 0.01879, p = 0.9814, sex difference: F[1,54] = 0.4936, p = 0.4854; interaction: F[2,54] = 0.7540, p = 0.4754; n = 10 per group), the number of entries in the centre of the arena (2-way ANOVA; Shift-work paradigm effect: F[2,54] = 1.482, p = 0.2362, sex difference: F[1,54] = 2.155, p = 0.1479; interaction: F[2,54] = 1.455, p = 0.2424; n = 10 per group), the latency to the first entry in the centre of the arena (2-way ANOVA; Shift-work paradigm effect: F[2,54] = 1.494, p = 0.2335, sex difference: F[1,54] = 0.1239, p = 0.7262; interaction: F[2,54] = 4.689, p = 0.0133; n = 10 per group), the total distance travelled (2-way ANOVA; Shift-work paradigm effect: F[2,54] = 3.098, p = 0.0533, sex difference: F[1,54] = 0.6401, p = 0.4272; interaction: F[2,54] = 0.2903, p = 0.7492; n = 10 per group), nor the average speed (2-way ANOVA; Shift-work paradigm effect: F[2,54] = 3.126, p = 0.0519, sex difference: F[1,54] = 0.8041, p = 0.3739; interaction: F[2,54] = 0.3034, p = 0.7396; n = 10 per group).

### 3.3 Startle reflex and prepulse inhibition

The acoustic startle response and the prepulse inhibition responses at 70 db or 75 db were not significantly altered by either sex or shift work paradigm (startle response: 2-way ANOVA; shift-work difference: F(2,45) = 0.06, p = 0.944; sex difference: F(1,45) = 1.95, p = 0.169; interaction: F(2,45) = 0.24, p = 0.785. PPI at 70 db: 2-way ANOVA; shift-work difference: F(2,45) = 0.2, p = 0.816; sex difference: F(1,45) = 0.74, p = 0.394; interaction: F(2,45) = 0.43, p = 0.651. PPI at 75 db: 2-way ANOVA; shift-work difference: F(2,45) = 0.81, p = 0.452; sex difference: F(1,45) = 2.05, p = 0.159; interaction: F(2,45) = 1.32, p = 0.277. N = 7 for male cohorts, 10 for female cohorts).

### 3.4 Species-specific digging behavior

#### 3.4.1 Marble burying test

Neither shift-work nor the sex affects the number of marbles buried in the marble burying test (2-way ANOVA; shift-work difference: F[2,39] = 0.8035, p = 0.5567; sex difference: F[1,39] = 0.3515, p = 0.4550; interaction: F[2,39] = 0.02870, p = 0.9717, n = 5 and 10 per groups).

### 3.5 Correlations between circadian disruption and behavioural phenotypes

Since our dataset provided both circadian and behavioural data for each mouse analysed, we performed correlation analysis to establish whether circadian disruption is correlated with behavioural changes at an individual level. Pearson Correlations were performed upon our dataset, using Benjamini-Hochberg procedure for multiple test corrections and sex used as a categorisation factor. We then specifically identified correlations between circadian parameters measured during the shifting lighting conditions and behavioural parameters recorded in subsequent phenotyping tests. This analysis identified a significant correlation between the interdaily stability of animals during disruption and the latency to first enter the centre of the open field, exclusively in female animals (Table 2, Fig. 3A). Additionally female animals also showed a near significant correlation between the amplitude during the disruption period and the latency to first enter the centre of the open field (Table 2, Fig. 3B). Notably no significant or near significant correlations between circadian and behavioural parameters were identified in male animals.

**Table 2:** Significant correlations between circadian disruption and specific behavioural parameters in female animals. IS: interdaily stability; vs: versus.

First variable		Second variable	n	Correlation Coefficient	Test statistic	Corrected p-value
IS during disruption	vs.	Open Field: latency to first entry to centre	30	0.476	2.865	0.0312
Amplitude during disruption	vs.	Open Field: latency to first entry to centre	30	0.395	2.275	0.0616

## 4 Discussion

### 4.1 Photoperiod shifts

Misalignment of light-dark cycles with the internal circadian rhythm of the individual or model organisms are a commonly used method to induce and study the effects of circadian disruption (see for example McGowan and Coogan (2013), Okuliarova et al. (2016)). While such studies have successfully characterised the consequences of circadian misalignment to an organism’s health or behavior, the effect of the disruption schedule on the affected model organism over the course of the disruption is often relatively uncharacterised. It has been noted that a range of different lighting schedules has been used to induce circadian disruption and that different protocols can induce different downstream consequences(Fisk et al., 2018). Using a number of circadian parameters, we have characterised the direct circadian effects of the altered light cycles described here upon mice using running wheels. Here we show that under disruptive conditions, mice show a dampening of circadian rhythmicity by Lomb-Scargle periodgram analysis. Further analysis using non-parametric variables of rhythmicity(Van Someren et al., 1999) show that this dampening is due to poor synchronisation between the light and activity rhythms across subsequent days (shown by a reduction in interdaily stability (IS) in disrupted animals) rather than a more disrupted rhythm within the day (show by a lack of change in the intradaily variability (IV) in disrupted animals). It is notable that while changes in IS and IV have been used to characterise changes in circadian rhythms due to aging(Banks et al., 2015; Huang et al., 2002) or neurodegenerative diseases(Anderson et al., 2009; Hatfield et al., 2004), we are unaware of studies which have characterised the consequences of induced changes specifically in either IS or IV. Subsequent analysis of the disrupted animals in this study (discussed below) suggests that changes in IS alone are likely to have mild consequences at a behavioural level.

### 4.2 Behaviour and shift-work paradigms

Our data do not support result of McGowan and Coogan (2013) who observed an increase of the activity in the alternate shift-worked mice compared to the control mice and an increase of anxiety-like in the backward and alternate shift-worked mice in the open field. While we induced the same shift-work paradigm, our methodology differs in few points. We did not expose our mouse to two-week constant darkness after completion of the paradigm shift, we rather exposed them to the same 12-h light-dark condition than the control for two weeks. We also used both female and male inbred mouse strain instead of albino male outbreed only. Both methodology could explain the contrasting results as light condition and the present or lack of retina pigmentation impact on mice behavior(Alves-Simoes et al., 2016), also albino mice are more sensitive to light(La Vail et al., 2009). However, the effect of light condition such as constant darkness or 12-h light-dark do not seem to affect the stress level assessed by plasma corticosterone in B6J or CD-1 mice(Alves-Simoes et al., 2016), however they have not tested stress-like behaviors in mice. Finally, we performed a wider range of behavioral tests. Because mice are social animal, we have transferred the singled house female mice to group housing at the end of the photoperiod shift. We were not allowed to do the same for male mice that has been singled house for an extend period of time due to potential aggression behavior between male mice. Therefore, our sex different observed in the light-dark box test could have been the results of the re-housing rather than a sex difference. Studies on the behavioral effects of housing conditions have been done with different outcomes such as behavioral differences observed in male C57BL/6 J (Võikar et al., 2005) including following changed in housing conditions and type of cage(Pasquarelli et al., 2017) but not in female and male C57BL/6NCrl(Arndt et al., 2009). Additionally, our correlation analysis shows that circadian disruption in female animals is correlated with the latency to enter the centre of the open field. In concordance with our result, Okuliarova et al. (2016) have shown a reduction of light aversion in male rats exposed to repeated light shift exposure. Because there is no other difference observed in hyperactivity and other anxiety-like parameters measured in female and male groups, our results could suggest a reduction of light aversion in male mice and an increase of exploration initiative in female compared to male, however more studies are necessary to investigate this hypothesis. Interestingly both female and male mice subject to alternated shift display a reduction of voluntary movement. We did not assess metabolism factors in these mice, but further study would be necessary to investigate whether the reduction of wheel running activity found in our alternated shifted mice is associated with metabolism dysfunctions and obesity(Karlsson et al., 2001). However, Saderi et al. (2019) has previously shown locomotor activity, and behavioral and molecular metabolic-associated factors to recover at respectively day 2, day 6 and day 7 after a protocol of shift-work in male rats(Saderi et al., 2019), therefore we would not expect substantial changes in metabolism factors in our experiments after 2 weeks of re-entrainment from shift-work-like patterns.

### 4.3 Caveats of modelling human shift work in mice

The protocol to study shift-work in mice consists of a photoperiod shift with a clear light and a dark phase that is unlikely present in shift-workers daily life. In addition, shift-work does affect psychological and social aspects. Indeed, being out of phase with the society, friends and family influences the wellbeing and quality of life of the shift-workers(Costa, 2003; Loudoun, 1997). Whereas social aspect is important in mice, our mice were singly housed during the shift-work paradigm. The current study allowed to focus on specific light-shift paradigm effects on anxiety-like behaviour however for a closer model of human shift-work further investigation are needed. For instance it would be interesting to measure the anxiety-like and social aspects of the shift-worked mice housed with non-shift-worked mice using the forced-activity during light time shift work paradigm(Salgado-Delgado et al., 2010).

## 5 Conclusion

For a better representation of the population, we have assessed the effect shift-work-like pattern on behaviors of both female and male mice. We mostly found no behavioural changes in mice that underwent shift-work paradigm, confirming a recovery effect as previously seen in male rats(Saderi et al., 2019). However, we have shown that only the alternated shift reduces voluntary movement activity of both male and female mice, and only the forward shift reduces light aversion of male mice. We also have shown that despite seeing an altered weekly rhythm stability of the shift-worked mice, their daily variability rhythms do not change along the weekly cycle. This daily rhythm stability maintained over the weeks of repeated shift-paradigm could explain the mild effect of shift work-like patterns on mouse behaviors. Several studies have shown that the negative impact of circadian disruption depends upon the degree to which an animal is able to entrain to the disruptive conditions and that if mice can make some degree of adjustment to the shift-work conditions, the deleterious effects on health may be mitigated(Delorme et al., 2022; Inokawa et al., 2020). However, the correlation analysis we present here suggests that, at the level of the individual animal, certain behaviors may still be impacted by previous periods of circadian disruption. Notably we highlight sex differences in these correlations, with the correlations we describe being exclusive to females. Further work is necessary to establish how long lasting such correlations are and whether other aspects of behaviour and physiology are modulated in the same way. Finally, we shown that in non-disruptive condition, female mice have a stronger daily rhythm and a higher voluntary activity compared to male.

## CRediT authorship contribution statement

**Gareth Banks:** Conceptualization, Investigation, Methodology, Software, Data curation, Formal analysis, Writing – review & editing. **Patrick M. Nolan:** Funding acquisition, Resources, Writing – review & editing. **Nora Bourbia:** Conceptualization, Investigation, Methodology, Software, Data curation, Formal analysis, Project administration, Writing – original draft, Writing – review & editing, Funding acquisition, Supervision.

## Declaration of competing interest

None.

## Data Availability

Data will be made available on request.
